# Bioengineering the ameloblastoma tumour to study its effect on bone nodule formation

**DOI:** 10.1038/s41598-021-03484-5

**Published:** 2021-12-16

**Authors:** Deniz Bakkalci, Amrita Jay, Azadeh Rezaei, Christopher A. Howard, Håvard Jostein Haugen, Judith Pape, Shosei Kishida, Michiko Kishida, Gavin Jell, Timothy R. Arnett, Stefano Fedele, Umber Cheema

**Affiliations:** 1 UCL Centre of 3D Models Health and Disease, Division of Surgery and Interventional Sciences, University College London, Charles Bell House, London, UK; 2 University College London Hospitals, London, UK; 3 Division of Surgery and Interventional Sciences, University College London, Royal Free Campus, London, UK; 4 Deparment of Physics & Astronomy, University College London, London, UK; 5 Department of Biomaterial Institute for Clinical Dentistry, University of Oslo, Oslo, Norway; 6 Department of Biochemistry and Genetics, Graduate School of Medical and Dental Sciences, Kagoshima University, Kagoshima, Japan; 7 Department of Cell and & Developmental Biology, University College London, London, UK; 8 Eastman Dental Institute, University College London, London, UK

**Keywords:** Cancer models, Experimental models of disease

## Abstract

Ameloblastoma is a benign, epithelial cancer of the jawbone, which causes bone resorption and disfigurement to patients affected. The interaction of ameloblastoma with its tumour stroma drives invasion and progression. We used stiff collagen matrices to engineer active bone forming stroma, to probe the interaction of ameloblastoma with its native tumour bone microenvironment. This bone-stroma was assessed by nano-CT, transmission electron microscopy (TEM), Raman spectroscopy and gene analysis. Furthermore, we investigated gene correlation between bone forming 3D bone stroma and ameloblastoma introduced 3D bone stroma. Ameloblastoma cells increased expression of MMP-2 and -9 and RANK temporally in 3D compared to 2D. Our 3D biomimetic model formed bone nodules of an average surface area of 0.1 mm^2^ and average height of 92.37 $\pm$ 7.96 μm over 21 days. We demonstrate a woven bone phenotype with distinct mineral and matrix components and increased expression of bone formation genes in our engineered bone. Introducing ameloblastoma to the bone stroma, completely inhibited bone formation, in a spatially specific manner. Multivariate gene analysis showed that ameloblastoma cells downregulate bone formation genes such as *RUNX2*. Through the development of a comprehensive bone stroma, we show that an ameloblastoma tumour mass prevents osteoblasts from forming new bone nodules and severely restricted the growth of existing bone nodules. We have identified potential pathways for this inhibition. More critically, we present novel findings on the interaction of stromal osteoblasts with ameloblastoma.

## Introduction

Ameloblastoma (AM) is a benign odontogenic epithelial tumour consisting of nests of neoplastic cells within the jawbones that resemble enamel-forming organs, but do not differentiate further to deposit enamel^1,2^. Ameloblastoma is an aggressive, locally invasive tumour causing bone resorption^3^. Ameloblastoma has a high potential for local recurrence with the rates dependant on the type of surgical procedure, resulting in multiple surgical interventions including loss of function, and psychological burden^4,5^. Difficulties in diagnosis and treatment can contribute to recurrence of ameloblastoma^6,7^. Histologically, most AMs display a follicular or plexiform pattern characterised by islands of epithelium with columnar, preameloblast-like, palisaded cells with reverse polarised nuclei lining the basement membrane and superficial layers of loosely arranged cells, resembling stellate reticulum of the cap /bell stage of a developing tooth^8,9^.

A range of gene mutations and copy number alterations have been identified and suggested as potential drivers of AM pathogenesis, mostly within the MAPK cascade pathway such as *BRAF* V600E, FGF2 and RAS^10–12^. Notably less research has been conducted concerning the mechanisms driving bone remodelling in the microenvironment surrounding the neoplastic cells, even though AM growth typically causes bone resorption^4,5^.

Normal tooth development, including initiation, morphogenesis and eruption, involves bone remodelling, osteoclastogenesis and cross-talk between odontogenic epithelium and the surrounding bone cells^13,14^. Alveolar bone resorption occurs during tooth eruption and osteoclastogenesis is driven via the receptor activator of nuclear factor kappa B-ligand (RANKL) and bone morphogenic-protein (BMP-2)^14^. Parathyroid hormone related protein (PTHrP) regulates dental eruption by the activation of osteoclasts around the dental germ and causing osteolysis^15^.

Bone resorption occurring in tooth eruption has similarities with AM invasion into the surrounding bone. AM research has focused on how this tumour enhances the active recruitment of osteoclasts to degrade/breakdown bone^16^. The current theory is that AM increases RANKL, which then binds to its receptor RANK on the surface of osteoclasts and causes osteoclast activation and thereby, bone resorption^17,18^ Strong PTHrP expression in AM is also involved in the activation of osteoclasts^19^. The matrix metalloproteinases (MMPs) in particular MMP-2 and -9 found in AM epithelium cause further osteoclasts activation as well as degradation of fibrillar collagen (collagen type IV)^20,21^.

The above studies include immunohistochemistry on ex-vivo specimens and two-dimensional (2D) cell cultures with primary and immortalised AM cell lines^22,23^. However, three-dimensional (3D) models including spheroids^24^, hydrogels^25^, organoids^26^, and tumouroids^27^ are known to provide deeper insight into the relationship between tumours and their native microenvironment^28^. There are only a few proposed 3D ameloblastoma models. Fuchigami and co-workers developed a 3D organotypic soft tissue model of fibroblasts and AM cells, and demonstrated that fibroblasts can potentiate collective cellular invasion form of AM cells^29^. Eriksson et al., reported higher RANKL expression by the AM cell line (AM-1 immortalised from plexiform type) when they were cultured with a human osteosarcoma (HOS) cell line and decellularised bone granules in a 3D organotypic model^30^. Recent work by Lee et al*.*, showed a decrease in mineralisation by mouse osteoblast cells (ST2) when they were cultured with AM-1 in 3D, which in turn increased the proliferation of the AM-1 cells^31^.

Nevertheless, none of the above ameloblastoma 3D models included an active bone-forming stroma, which represents the native tumour microenvironment of AM and arguably the most appropriate and biomimetic organotypic model. Developing a biomimetic active-bone forming model is challenging, with current 3D bone tumour models being limited to features such as mineral deposition^32^ or deposition of bone nodules^33^.

In our study, we have developed an active bone-forming 3D model with detailed characterisation. We wanted to undertake extensive characterisation of 3D in vitro bone nodule formation in dense collagen matrices, to match, at a minimum, that done for 2D bone nodule formation, including mineralisation and gene markers. For confirmation of definitive in vitro bone nodule formation it was paramount to measure structure and composition as well as to investigate of specific gene panels for all stages of bone formation^34–36^.

We report our work on the development and utilisation of 3D tumouroids, which are composed of dense collagen^27^ to generate tumour-stroma models for AM where AM cell lines AM-1 (plexiform)^37^ and AM-3 (follicular)^21^ are cultured within biomimetic bone stromal compartments containing osteoblasts, which actively form dense bone nodules. We investigated how the AM cells interfere with osteoblast-led bone formation in order to suggest novel mechanism of disruption of bone homeostasis associated with AM.

## Materials and methods

### Cell culture

All cultures were maintained in 37 °C, 5% CO_2_, and 21% O_2_ atmospheric pressure at all times. AM-1 cells were immortalised from plexiform ameloblastoma and kindly provided by Prof H. Harada^37^. AM-1 cells were cultured in keratinocyte serum free medium 1X (KSFM) supplemented with KSFM supplements (bovine pituitary extract (BPE) and EGF, human recombinant). AM-3 cells were immortalised from follicular ameloblastoma and kindly provided by Prof Kishida and colleagues from Kagoshima University, Japan^29^. AM-3 cells were cultured in defined KSFM with DKSFM supplement. MG-63 osteosarcoma cells were obtained from European Collection of Authenticated Cell Cultures (ECACC). MG-63 cells were cultured in Dulbecco’s modified Eagle medium (DMEM). All media types were also supplemented with 10% Foetal bovine serum (FBS), 100 units/mL penicillin and 100 μg/mL streptomycin (Gibco through Thermo Fisher Scientific, Loughborough, UK).

### 3D models

Monomeric type 1 collagen (First Link, Birmingham, UK) was used to fabricate all 3D tumouroids. Collagen hydrogels were plastically compressed using the RAFT protocol (pages 3–10) (Lonza, Slough, UK) for collagen hydrogel preparation, which was kept on ice and sterile^27^. Initially, 10X Minimal Essential Medium (MEM) (Sigma-Aldrich, Dorset, UK) was mixed with collagen and neutralizing agent (N.A.). The N.A. was prepared by combining 17% 10 Molar (M) NaOH (Sigma-Aldrich, Dorset, UK) and 83% 10 M HEPES buffer (Gibco through Thermo Fisher Scientific, Loughborough). After adding the cell suspension to the mix, the final volumes were 80% collagen, 10X MEM, 6% N.A. and 4% cells.

**Figure 1:**
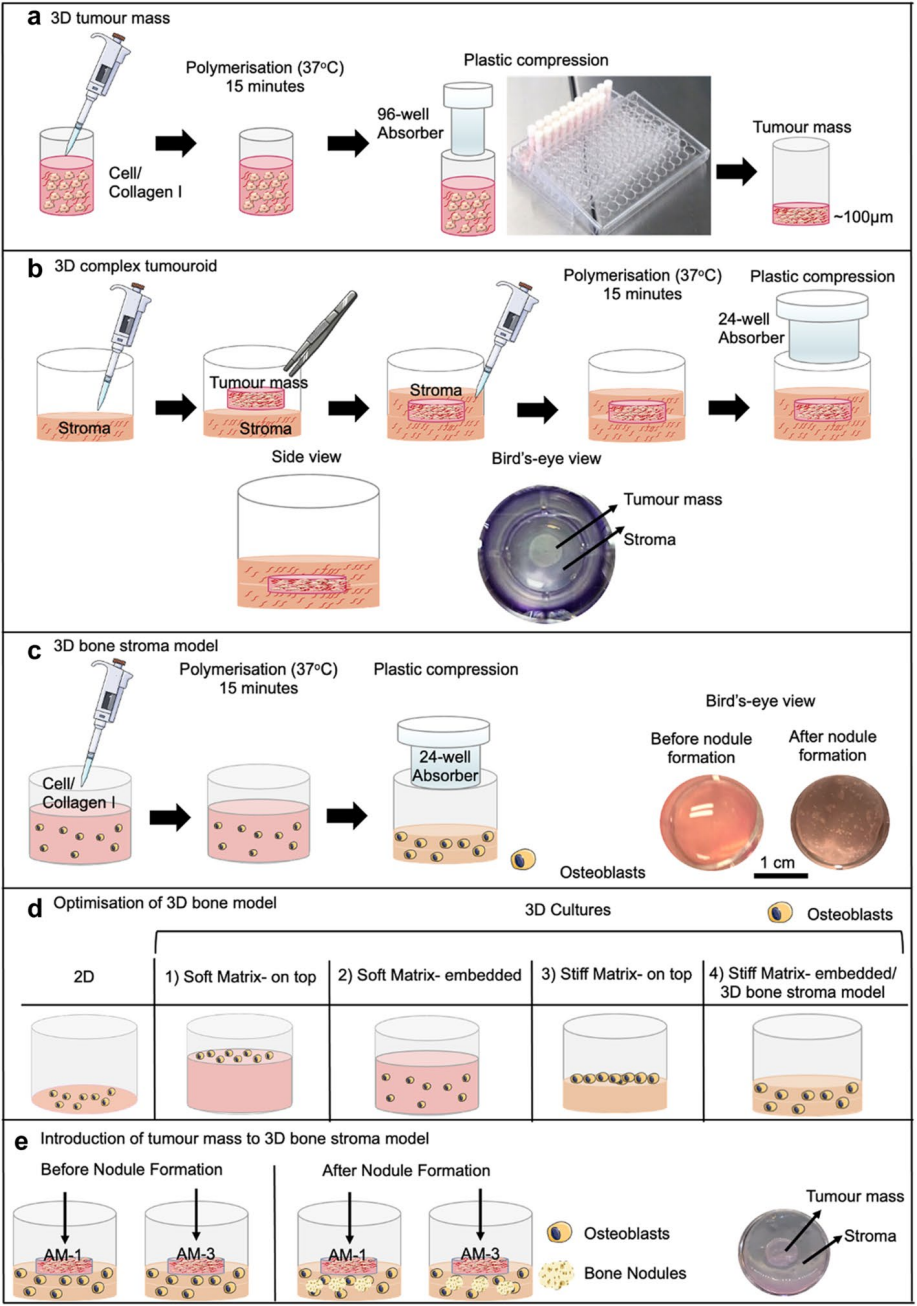
Establishing 3D tumour models. (**a**) Fabrication of a tumour mass. (**b**) Complex tumouroids. (**c**) 3D bone stroma model. (**d**) Different set-ups and matrices used for the optimisation of 3D bone stroma model. (**e**) Introduction of tumour masses to the 3D bone stroma model. Diagram was created using Servier Medical Art.

Each tumour mass was generated by setting a 240 μl of cell/collagen mix (5 × 10^4^ cells) into 96-well plates (Corning Costar, Sigma-Aldrich, Dorset, UK). The gel mix was set at 37 °C for 15 min and plastic compressed for 15 min to remove excess fluid using the RAFT absorbers at room temperature (Lonza, Slough, UK) (Fig. 1a).

The complex tumouroids were composed of a stromal compartment with tumour mass embedded within or placed on top. The stromal compartment was either acellular or contained primary rat calvarial osteoblasts. For embedded cultures, initially 650 μl of the gel mix was cast on 24-well plate (Corning Costar, Sigma-Aldrich, Dorset, UK) and let it sit for 5 min. tumour mass was placed in the middle of the first layer of the gel, which was followed by the application of the second stromal layer for a total of 1.3 ml. Tumouroids were left to undergo spontaneous fibrillogenesis for 15 min at 37 °C and then 24-well RAFT absorbers (Lonza, Slough, UK) were used for plastic compression for (15 min) at room temperature (Fig. 1b). 1 ml of media was applied per well with a 50% media change in every 48 h. The cultures were maintained for 21 days.

### 3D osteoblast culture

The calvarial rat osteoblasts were obtained and passaged as described in^38,39^. The cells were seeded either in 2D 24-well plates (control) or 3D hydrogels (uncompressed) or plastic-compressed (PC) collagen in 24-well plates. 3D cultures were prepared as described in the previous Sect. 1.3 ml of either collagen only or collagen/cell mix containing 7 × 10^4^ cells per well were left to polymerise for 15 min at 37 °C followed by 15 min plastic compression using 24-well absorbers RAFT absorbers (Lonza, Slough, UK) (Fig. 1c).

For the optimisation of 3D bone nodule formation, different culture conditions were prepared through using either uncompressed gels (soft hydrogel matrix, 0.2% collagen), or compressed gels (stiff matrix, 10% collagen)^40^. The osteoblasts were seeded either on top or mixed/embedded into different matrices (Fig. 1d) The culture medium was α-MEM (Gibco through Thermo Fisher Scientific, Loughborough, UK), supplemented with 10% FBS, 2 mM L-glutamine (Life Technologies) 1% antibiotic/antimitotic (100 units/ml penicillin, 100 μg/ml streptomycin, 0.25 μg/ml amphotericin) (Sigma-Aldrich, Dorset, UK) with half a media change (500 μl) every 48 h. Both 2D and 3D cultures were cultured for 3 days before the application of α-MEM with bone morphogenic agents (BMA). For BMA preparation, α-MEM was supplemented with 2 mM β-glycerophosphate, 10 nM dexamethasone, and 50 μg/ml ascorbate (Sigma-Aldrich, Dorset, UK). Depending on the experiment, the cultures were maintained for up to 21 days.

All methods were carried out in accordance with relevant guidelines and regulations. All experimental protocols were approved by the University College London Biological Services Ethical Review Committee and licensed under the UK Home Office regulations and the Guidance for the Operation of Animals (Scientific Procedures) Act 1986 (Home Office, London, United Kingdom). The study was carried out in compliance with the ARRIVE guidelines (http://www.nc3rs.org.uk/page.asp?id=1357).

### Introduction of tumour masses to the 3D bone stroma model

Tumour masses with either 5 × 10^4^ AM-1 or AM-3 cells were placed on top of the 3D bone stroma model at days either 3, 6, 9, 12, 15, or 18. At each time point, fresh tumour masses were prepared and 900 μl of media was removed from 3D bone stroma model cultures. The tumour mass was gently placed on top the 3D bone culture through a tweezer and then incubated for 10 min at 37 °C for the attachment of tumour mass to the 3D bone stroma model (Fig. 1e). The migration of cells was observed from the tumour mass into the bone stroma and vice versa. 1 ml of α-MEM with BMA was added slowly from the side of the well and 50% media change was completed every 48 h.

### Characterisation of bone nodules

**Figure 2:**
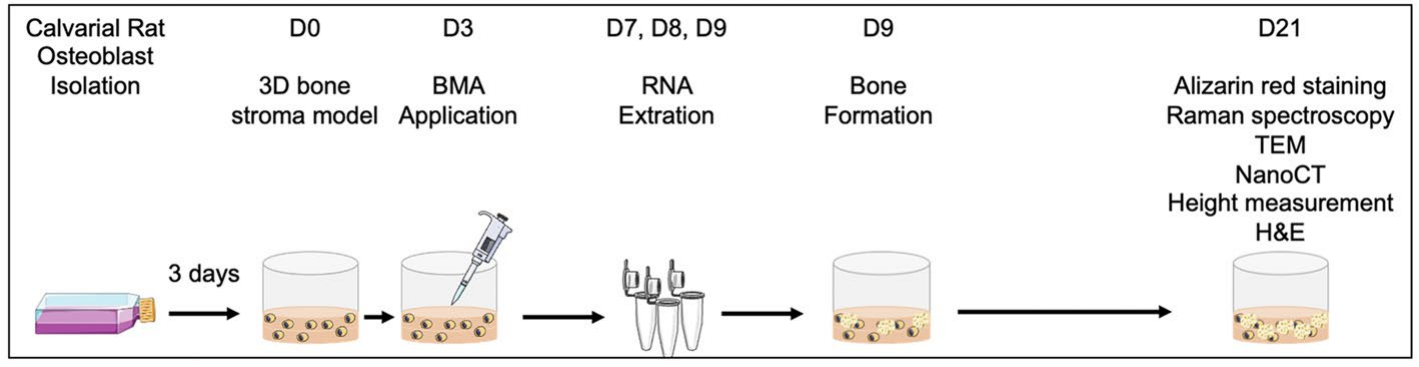
Time points for each bone nodule characterisation method. Diagram was created using Servier Medical Art.

The experimental timeline for bone nodule formation and bone nodule characterisation was described in (Fig. 2). Initial assessment was ‘the visibility by eye under the microscope’. Through brightfield filter, black dots were defined as mineralisation based on the classification of Orriss et al*.*^41^. To avoid confusion due to the high calcium phosphate deposition, only nodules with sharply defined margins were considered as bone nodules. The brightfield images were taken by either the EVOS XL Core Confocal Microscope (Life Technologies) or the Zeiss AxioObserver with Apotome.2 instrument and software (Zeiss, Oberkochen, Germany). For height measurement, day 21 3D bone stroma model samples were air-dried on a Petri dish for 24 h. The heights of the bone nodules were measured relative to the baseline measurement using the Keyence VHX-7000 Digital Microscope (Keyence, Osaka, Japan).

#### Alizarin red staining assay

The Alizarin red staining (Sigma-Aldrich, Dorset, UK) was used to stain calcium at days 14 and 21. Upon formalin-fixation (Genta Medical, York, UK), the samples were incubated with 40 mM alizarin red stain for 20 min for 2D and 30 min for 3D. 3D samples require more ddH_2_O washes (minimum 5) than 2D samples. Images were captured via Nikon brightfield reflected light microscopy using a Nikon ‘Labophot’ 2A microscope, with 100 W epi-illumination and metallurgical objectives.

#### Raman spectroscopy

Raman spectroscopy was conducted on day 21 3D bone samples that were air-dried on 15 mm diameter. 1 mm thick magnesium fluoride (MgF_2_) discs (Crystan) for 24 h. Raman spectra were collected using an inVia spectrometer (Renishaw, Gloucestershire, UK) interfaced using a Leica microscope fitted with a 785 nm laser, laser spot size of ~ 2 μm and the integration time was 20 s. Raman measurements were taken from 9 different nodules from 3 different bone cultures with 20 × lens objective over wavenumber range of 400 to 1800 cm^-1^ and the instrumental resolution was ~ 2 cm^-1^. The top parts of bone nodules were chosen for the consistency of measurement. The background signal from 3D collagen was captured by measuring 9 collagen-only spots (Supplementary Fig. 1). The Raman spectra analysis was conducted based on Gentleman et al.,’s protocol^42^. MATLAB ‘Raman Baseline Correction’ code (The Mathworks, MA, USA) was used to remove the average background signal from the 3D bone sample readings. The bone quality assessed by calculating degrees of mineral crystallinity from the full width of the sample at half maximum (FWHM) of the phosphate peak (PO_4_^3-^$\nu$_1_). The mineral to matrix ratio was calculated by dividing the phosphate band area by the matrix band area (amide I).

#### Transmission electron microscopy (TEM)

Samples were fixed in 2.5% Glutaraldehyde overnight followed by 2% EDTA for decalcification overnight. The first step was 100% ethanol dehydration of the samples and then infiltration procedure of 1-h Agar 100 epoxy resin mix, 1-h propylene oxide and 4 h pure epoxy resin. Medium sized bone nodules were chosen and cut trans sectionally for ~ 70 nm (nm) sections through a Reichert ultra-cut S microtome with a diamond knife (Leica, Milton Keynes, UK). A JEOL 1010 transition electron microscope (TEM; Tokyo, Japan), operated at 120 kV, was used for imaging of the section.

#### Nano-computed tomography (Nano-CT)

The 3D bone nodules were air-dried for 24 h, and the nodules were separated from collagen using tweezers (TAAB Laboratories Equipment Ltd, Aldermaston, UK). Each nodule was cut into smaller pieces and placed inside a 1 mm Kapton tube (DuPont, Shanghai, China). All specimens were scanned by a nano-CT (SkyScan 2211 Multiscale X-ray Nano-CT Sytem, Bruker micro-CT, Kontich, Belgium) with a 20–190 kV tungsten X-ray source and a dual detection system: an 11- megapixel cooled 4,032 × 2,670-pixel CCD-camera and a 3-megapixel 1,920 × 1,536 pixel CMOS flat panel. The specimens were scanned at 60 kV, 320 μA and 1000 ms over 360^o^ with a rotation step of 0.31°, leading to a final voxel size of 250 nm. The scan duration for samples was about two hours. Nano-CT projections were reconstructed using the system-provided software. NRecon (version 1.7.4.6) with smoothing kernel 2, ring artefact correction 9, and beam hardening correction of 20%. The 3D image sets were visualised with CTAn (Bruker micro-CT, Kontich, Belgium, version 1.18.4.0).

### RNA extraction, cDNA synthesis, qPCR (quantitative polymerase chain reaction)

For RNA extraction TRI Reagent was used for phase separation followed by the chloroform method^43^. For each condition, a minimum of 3 replicates were extracted. Two 3D samples were pooled unlike individual 2D culture extraction in order to maximise RNA quality and quantity, which was tested via Nano-Drop. High-Capacity cDNA Reverse Transcription Kit (Applied Biosystems through Fisher Scientific, Loughborough, UK) was used to transcribe RNA into cDNA using the T100 Thermal Cycler (Bio-Rad, Watford, UK). Minimum Information for Publication of Quantitative Real-Time PCR Experiments (MIQE) guidelines was followed during designing of primer pairs^44^ Supplementary Table 1. The primer conditions were presented in the Supplementary Table 2. The annealing temperature was set to 60 °C and the primer pairs were obtained from Eurofins Genomics (Ebersberg, Germany). The iTaq Universal SYBR Green Supermix was used to amplify the target gene as in 10 µl reactions composed of 20 ng sample and 0.2 µM primer concentration. The reaction was run for 40 cycles on the CFX96 Touch System (both from Bio-Rad, Watford, UK). The ∆CT and 2^-∆∆CT^ method^45^ was used to analyse the relative gene expression normalised to the reference genes for rat osteoblasts *Glyceraldehyde 3-phosphate dehydrogenase (GAPDH)*^46^ and for ameloblastoma cell lines *hypoxanthine–guanine phosphoribosyltransferase (HPRT1)*^47^.

A Rat Osteogenesis RT2 Profiler PCR Array (96-well format) Rat Osteogenesis Cat. No. 330231 PARN-026ZA (Qiagen) was used to investigate the effect of the introduction of tumour mass of ameloblastoma on bone formation by osteoblasts in the 3D bone stroma Supplementary Table 3. Samples from day 8 of the 3D bone stroma model. 3D bone stroma + AM-1 tumour mass, or 3D bone stroma + AM-3 tumour mass was lysed as described above and 500 ng/μL of RNA was extracted per sample. For the array, AM tumour masses were cast on day 6, and RNA extraction was completed on day 8. For this step, tumour masses were removed from the 3D bone part to minimise contamination from AM cells. RNA was processed via RNeasy Kit (Qiagen) and RT2 first strand kit (Qiagen) was used for cDNA transcription. Real-time PCR was conducted upon manufacturer’s instructions for RT2 SYBR Green qPCR Mastermix (Qiagen) in a Bio-rad CFX96 PCR system (Biorad). With three plates per condition, a total of 9 plates were used. CT values were submitted to RT2 PCR array data analysis (Qiagen) (www.qiagen.com/geneglobe). Fold changes were calculated using 2^-∆∆CT^ method. Each test group was compared to the control group and fold changes > twofold with p-value < 0.05 was accepted as significant. Student’s t-test was used to determine p-values of the replicates of 2^-∆∆CT^ values.

### Imaging and measurement of invasion

All 3D samples were imaged via either the Zeiss AxioObserver with Apotome.2 instrument and software (Zeiss, Oberkochen, Germany) or the EVOS XL Core Confocal Microscope (Life Technologies) based on a previously described method^48^. Cancer cells clustering as spheroids were defined as spheroids and outgrowth of cancer cells from tumour mass boundary towards the surrounding stroma was defined as ‘invasion’. Invasion distance was determined by the distance of the location of cancer cells from the tumour mass. All image analyses were completed by using ImageJ (NIH, USA) and data analyses by using GraphPad Prism 8 Software.

### Histology

3D samples were formalin fixed followed by processing of the samples in a processor (Thermo Fisher Scientific, Loughborough, UK), wax embedding and sectioning of samples using a microtome into 5 µm sections. The sections were placed on to glass slides for oven baking at 64 °C for 2 h. Manual haematoxylin and eosin (H&E) staining was then conducted following from cycles of xylene, alcohol and water washes before and after H&E staining. The mounting medium was applied before imaging via the Zeiss AxioObserver with Apotome.2 instrument and software (Zeiss, Oberkochen, Germany).

### Metabolic activity assays

The CellTiter-Glo 3D Viability-Assay (Promega, Southampton, UK) was mixed with media at a ratio of 1:1 and then incubated 5 min on a plate shaker and 25 min on the benchtop with light protection. Measurements were taken in triplicate using the Tecan Infinite Lumi plate reader (Männedorf, Switzerland). All values were normalised to media control readings.

### Immunofluorescence

10% neutrally buffered formalin (Genta Medical, York, UK) was applied for 30 min for formalin fixing of samples. This step was followed by 1-h permeabilization and blocking by 2% Triton-X 100 and 1% bovine serum albumin (BSA) (Sigma-Aldrich, Dorset, UK) at room temperature. BSA diluted primary antibodies anti-MMP-2 (ab97779), anti-MMP-9 (ab38898), anti-RANK (ab222215), anti-RANKL (ab45039) and anti-osteocalcin (ab134418) were added to samples for 1 h at room temperature (Abcam, Cambridge, UK). Then, BSA was used to dilute the secondary antibodies Goat Anti-Rabbit IgG H&L (Alexa Fluor 594) (ab150080) and Goat Anti-Mouse IgG H&L (Alexa Fluor 594) (ab150116). The secondary antibody was applied for 2.5 h at room temperatures based on the manufacturer’s protocols (Abcam, Cambridge, UK). The Samples were stained with Alexa Fluor 568 Phalloidin and counterstained with NucBlue, both from Invitrogen through Fisher Scientific, Loughborough, UK).

### Enzyme-linked immunoabsorbent assay (ELISA)

Cell culture supernatants were collected in triplicates. Total MMP-2 Quantikine ELISA Kit (MMP200, R&D Systems, Abingdon, UK), Human MMP-9 Quantikine ELISA Kit (DMP900, R&D Systems, Abingdon, UK) and Human TNFSF11/RANKL ELISA Kit PicoKine (EK0842, BosterBio, CA, USA) were used based on each of the manufacturer’s protocol. Measurements were conducted on the Tecan M200 PRO Microplate Reader (Männedorf, Switzerland).

### Statistical analyses

Statistical analyses were completed on GraphPad Software Inc., La Jolla, CA, USA. A minimum of 3 experimental repeats were considered for statistical analyses. Initially the normality of the data was tested by using a Shapiro–Wilk test (n $\ge$ 3) or D’Agostino test (n $\ge$ 8). Upon the normality test, appropriate statistical significance tests were used on the data, and the details are provided within the figure legends. The graphs are presented as mean $\pm$ standard error mean (SEM) and the text values as mean $\pm$ standard deviation (SD). Statistical significance was considered as p-value < 0.05.

## Results

### Characterisation of ameloblastoma cell lines in a 3D biomimetic model

**Figure 3:**
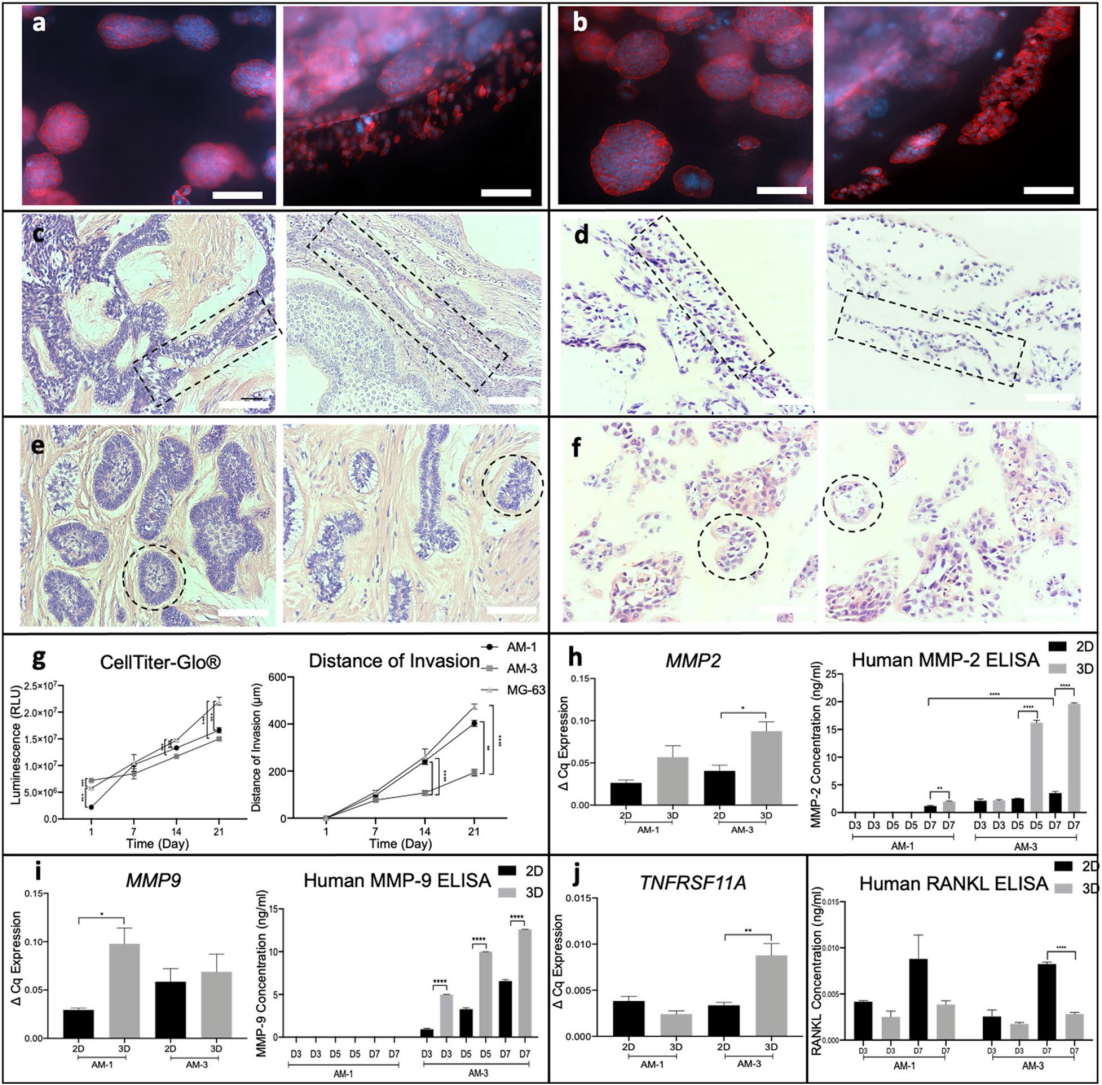
Characterisation of ameloblastoma cells in 3D culture. Spheroid formation of (**a)** AM-1 cells and (**b)** AM-3 cells in 3D tumouroids at day 7, red = Phalloidin, blue = DAPI, 20 × Magnification Scale Bar = 50 μm. (**c)** Histology H&E staining of plexiform patient samples, (**d)** 3D AM-1 tumouroids, **e** follicular patient samples, and **(f)** 3D AM-3 tumouroids. Similar anastomosing cords and branches were highlighted (**c, d)** 10 × Magnification Scale Bar = 100 μm. The formation of the odontogenic islands was highlighted (**e,f)**. (**g)** CellTiter-Glo 3D Viability-Assay of AM-1, AM-3 and MG-63 cells in 3D tumouroids. Distance of invasion (μm) AM-1, AM-3 and MG-63 cells from the tumour mass to the surrounding stroma within the 3D tumouroids. (**h)** Human MMP-2 ELISA. Expression of MMP2. (**i)** Human MMP-9 ELISA. Expression of MMP9. (**j)** Expression of TNFRSF11A (RANK). Human RANKL ELISA. One-Way ANOVA, Dunnet’s Post Hoc; p-values 0.05 = *, 0.005 = **, 0.0005 = *** and 0.00005 = ****.

Both AM-1 (Fig. 3a) and AM-3 (Fig. 3b) cells formed clusters/spheroids in 3D tumouroids similar to other previously reported tumours^48,49^. By day 7, both cell lines started to invade into the surrounding stroma from the tumour mass boundary. AM-1 cells invaded as cell sheets (Fig. 3a, right), where AM-3 cells invaded as the invasive spheroid bodies (Fig. 3b, right). Patient samples from each subtype were analysed for direct comparison of histopathological properties. Histology of AM tumouroids was similar to their corresponding ameloblastoma subtype. AM-1 tumouroids mimicked the anastomosing cords^50^ formed in plexiform patient samples (Fig. 3c and d). (Fig. 3d). AM-1 cells were aligned and formed branches in tumouorids (Fig. 3d), which was also observed in plexiform patients (Fig. 3c). AM-3 cells in tumouroids (Fig. 3f) presented as odontogenic islands^50^, which are representative invasive morphologies seen in patients presenting with the follicular subtype of ameloblastoma (Fig. 3e). For metabolic activity and invasion in 3D, a well-established cell line in 3D, the MG-63 cells were used as control^49^. The metabolic activity of both AM-1 and AM-3 cells increased over time (Fig. 3g), with AM-3 exhibiting lower metabolic activity at all time points (p = 0.005). Differences in invasion distance was visible by day 14. By day 21, AM-1 cells invaded to a distance of 402 $\pm$ 13 μm, significantly greater than AM-3 cells, 250 $\pm$ 14 μm. Both AM-1 cells (p = 0.0268) and control MG-3 cells (p-value < 0.0001) invaded longer distances compared to AM-3 (Fig. 3g). Invasion distances of ameloblastoma did not change with a biomimetic stromal matrix of demineralised bone, NuOss to the surrounding stroma, thus deviating from other tumour cell lines shown to be directly influenced by a bone matrix^49^ (Supplementary Fig. 2).

The invasive properties of ameloblastoma cells and their ability to resorb bone were investigated by assessing the expression of MMP-2, MMP-9, RANK, and RANKL^16^ prior to initiation of invasion. Membrane-bound MMP-2, which is an invasive marker for ameloblastoma^51^ was identified in both AM-1 and in AM-3 tumouroids (Supplementary Fig. 3). *MMP2* gene was found to be 2 times higher in AM-1 and AM-3 3D tumouroids (p = 0.03) compared to 2D (Fig. 3h). Pro- and active forms of MMP-2 were detected only in the culture medium of AM-3 tumouroids (Fig. 3h). AM-3 cells released significantly higher levels of MMP-2 in 3D tumouroids 5 ng/ml compared to 2D, 1 ng/ml at day 3 (p-value < 0.0001) as well as days 5 and 7 (Fig. 3h). *MMP9* gene was threefold upregulated in AM-1 3D tumouroids than 2D AM-1 cultures (Fig. 3i). The membrane-bound (Supplementary Fig. 3) and pro- and active- forms of MMP-9 were identified in both AM-1 and AM-3 tumouroids (Fig. 3i). As AM-1 cells did not release MMP-9 to the culture medium, protein levels for AM-1 were not included in the ELISA graph. AM-3 cells started releasing MMP-9 by day 3. MMP-9 levels were found to be 6 times higher in AM-3 3D tumouroids than 2D at days 5 and 7 (p < 0.0001). *TNFRSF11A* (RANK) expression was threefold higher in AM-3 cells in 3D tumouroids than in 2D at day 7 (p = 0.005) (Fig. 3j). Membrane-bound RANK was detected earlier in 3D than in 2D at day 3 in AM-3 cells (Supplementary Fig. 3). The amount of RANKL released to culture medium was lower in 3D cultures compared to 2D. AM-3 cells in had threefold higher RANKL in 2D, 8.2 × 10^–3^ pg/ml than in 3D 2.8 × 10^–3^ (p-value < 0.0001) (Fig. 3j). Membrane-bound RANKL was identified in AM-3 3D tumouroids (Supplementary Fig. 3).

### Bone nodules formed in 3D Stiff Matrix but not in 3D Soft Matrix

**Figure 4:**
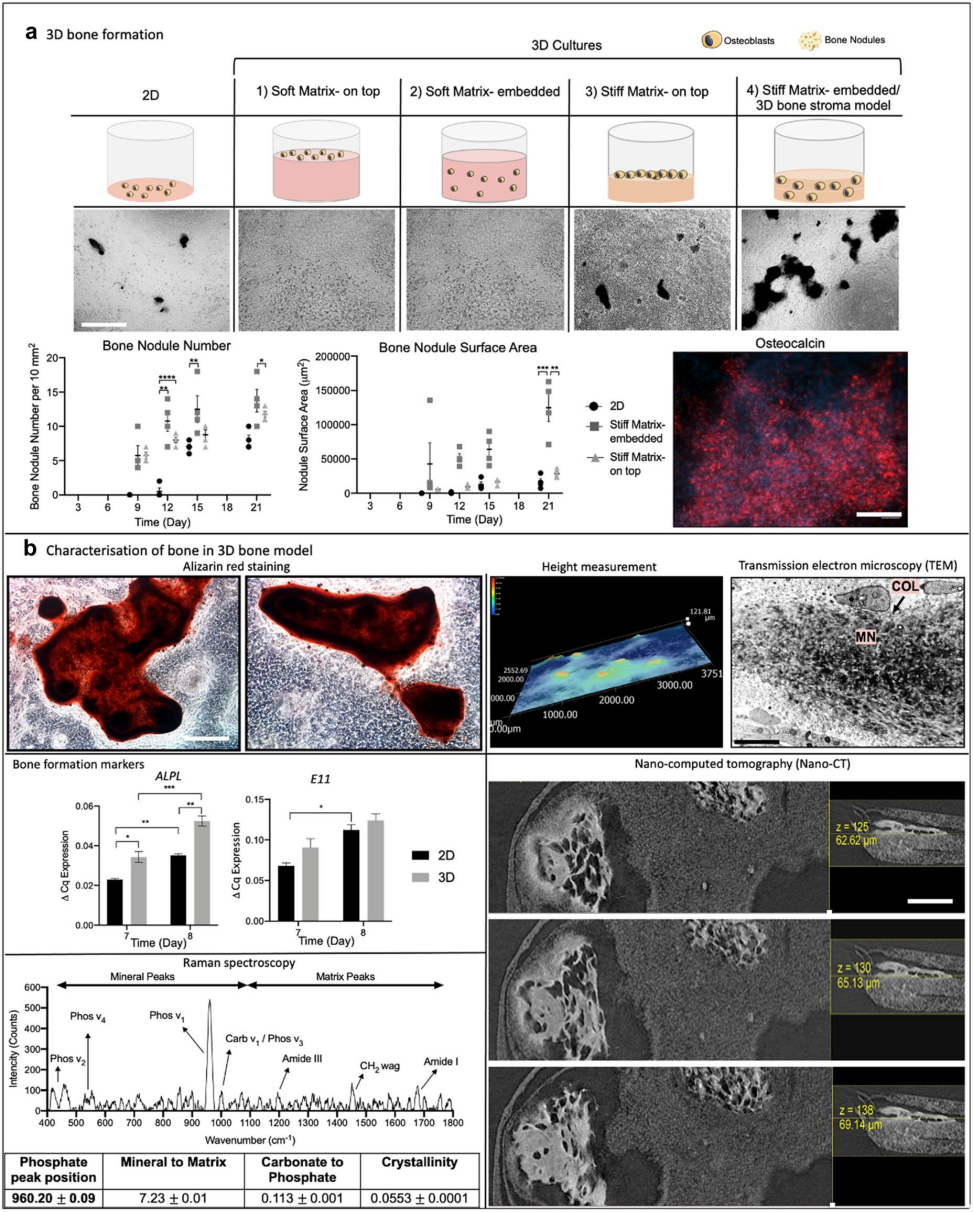
Characterisation of bone nodules produced in 3D culture. **(a)** Bone formation in 3D bone stroma model. Day 14 images, 4 × Magnification, Scale bar = 400 μm. The surface area of the bone nodules and the number of bone nodules produced in 2D, 3D stiff matrix-embedded and 3D stiff matrix- on top. Osteocalcin immunofluorescence at day 9, red = Osteocalcin, blue = DAPI, 10 × Magnification, scale bar = 200 μm. (**b)** Characterisation of bone nodules in 3D bone stroma model. Alizarin red stained nodules in 3D bone stroma model at day 21, 6 × Magnification, scale bar = 50 μm. Height Measurement of bone nodules in 3D bone stroma model at day 21. TEM images of the bone nodules formed in 3D bone stroma model at day 21 showing collagen fibrils and mineralised bone nodules, 10 × Magnification, scale bar = 4 μm. Expression of ALPL and E11 by the osteoblasts in the 3D bone stroma model at days 7 and 8. Screenshot of nanoCT scan video (Supplementary Video 1) of a bone nodule in 3D bone stroma model at day 21, scale bar = 100 μm. Raman spectra of the bone nodules in 3D bone stroma model at day 21. Table of intensity ratios calculated from the Raman spectra; values were mean $\pm$ SEM (f). One-Way ANOVA, Dunnet’s Post Hoc; p-values 0.05 = *, 0.005 = **, 0.0005 = *** and 0.00005 = ****.

We bio-engineered an active 3D model of bone. This was the active bone-forming stromal compartment containing osteoblasts, to which we could add a tumour mass of ameloblastoma to study its progression. Primary calvarial osteoblasts were cultured in different matrices for 21 days and directly compared to 2D control cultures. Culturing osteoblasts in soft collagen hydrogels resulted in osteoblasts mineralising the matrix, but no further bone nodule formation (Fig. 4a). We observed large nodule-like structures forming within the 3D stiff matrices where cells were embedded within the scaffold or cultured on top (Fig. 4a). Based on phase contrast images, 3D stiff matrix-embedded and 3D stiff matrix-on top formed bone nodules at day 9 compared to 2D culture on day 13. The scaffolds were transparent, and their thicknesses (approximately 200 μm) allowed for measurement of total nodules and nodule surface area using phase contrast microscopy. 3D stiff matrix-embedded cultures deposited the highest number of bone nodules per 10 mm^2^ at all time points and at day 21 significantly higher than that of 3D stiff- on top cultures (p = 0.05) (Fig. 4a). 3D stiff matrix-embedded cultures formed nodules 8 times bigger than 2D (p = 0.0005) and 4 times bigger than 3D stiff matrix- on top (p = 0.005) bone nodules (Fig. 4a). Thereby, 3D stiff matrix-embedded cultures were deemed as the optimal condition for in vitro bone nodule formation in 3D and hitherto referred to as the ‘3D bone stroma’ for the rest of the study. Bone nodules in 3D stiff matrix were additionally found to be positive for a late bone formation marker osteocalcin^52^ (Fig. 4a).

### Characterisation of bone nodules in the 3D bone stroma model

The bone nodules produced in the 3D bone stroma were verified by using well-established bone nodule characterisation methods. Alizarin red stain detected calcium within the bone nodules, including calcium dense areas (dark red) and less dense areas (light red) (Fig. 4b). The height of the bone nodules (z-axis) in the 3D bone stroma were measured. The average height was 92.37 $\pm$4 7.96 μm with a maximum height of 121.81 μm (Fig. 4b), in line with previously defined sizes of bone nodule (70–100 μm)^53,54^ (details of the height measurement were provided in the Supplementary Fig. 4). TEM imaging of the bone nodules shows dense collagen fibrils around the mineralised bone nodules (Fig. 4b). The osteoblast and mineralisation marker *ALPL*^55^ was higher in 3D compared to 2D at day 7 and 8 (p = 0.05, p = 0.005 respectively). The osteocyte marker *E11*^56^, was detected in the 3D bone stroma (Fig. 4b). The bone nodules were scanned from top to bottom and images were captured at different Z-stack sections of the nodules. The Nano-CT images of the bone nodules verified active and rapid bone formation upon detection of a woven structure throughout the bone nodule (Fig. 4b). The Raman active phosphate band at 960 cm^-1^ was used to determine the composition of the bone nodules from their Raman spectra. The Raman analysis showed there were peaks of the carbonate-substituted apatite and protein peaks associated with collagen similar to bone^42^ (Fig. 4b).

### Introduction of an ameloblastoma tumour mass inhibits or restricts bone nodule formation

**Figure 5:**
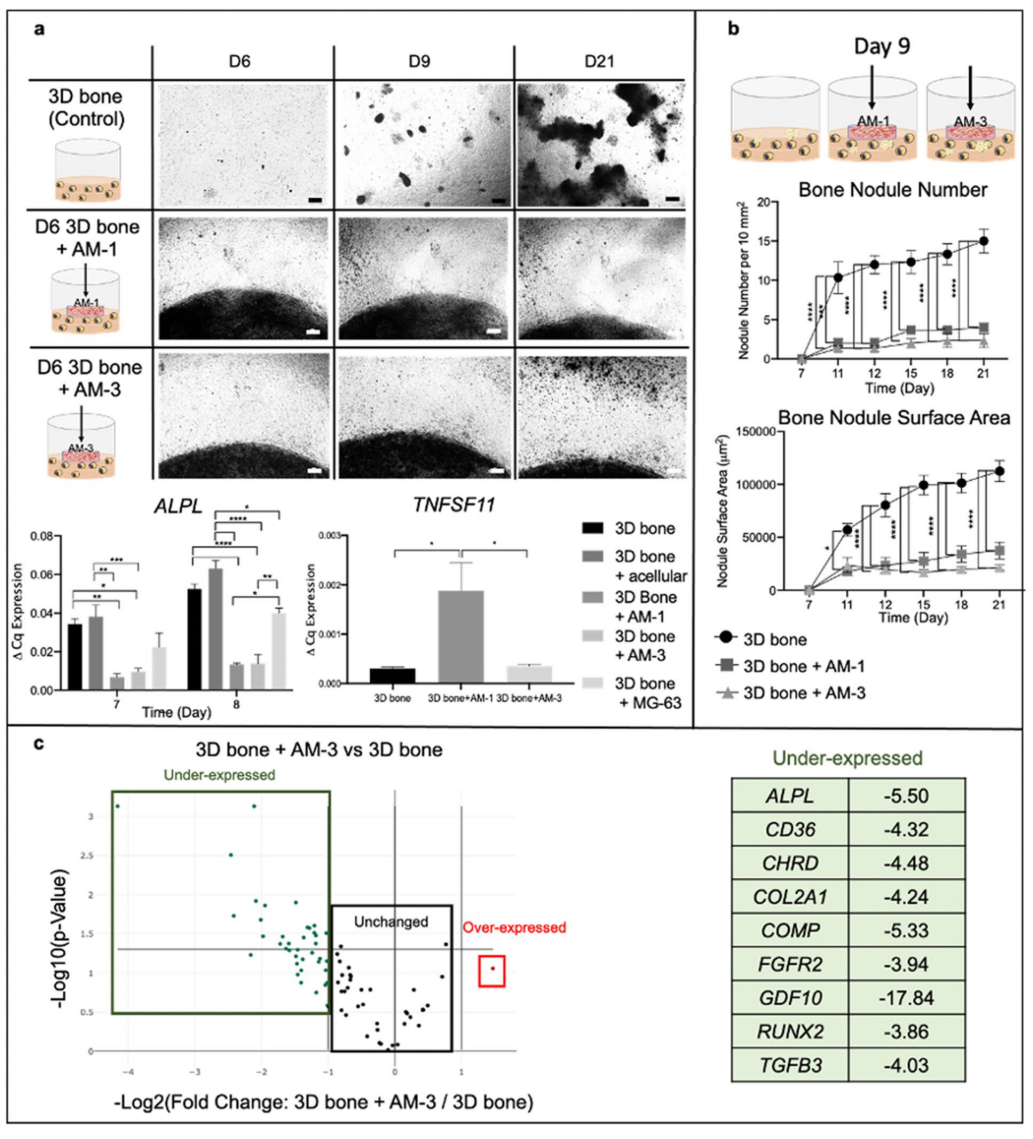
Introduction of ameloblastoma tumour mass to the 3D bone stroma prior to day 9 completely inhibits bone nodule formation. **(a)** Introduction of AM-1 and AM-3 tumour masses to 3D bone at day 6 inhibits bone nodule formation. Images taken at day 6, 9 and 21, 4 × Magnification, scale bar = 100 μm. ALPL expression. TNFSF11 (RANKL) Expression. (**b)** Introduction of AM-1 and AM-3 tumour masses to 3D bone at day 9 restricts bone formation by limiting bone nodule number and bone nodule surface area. **(c)** RT2 Profiler PCR Array was conducted to screen osteogenesis gene of osteoblasts in the 3D bone stroma model and in AM-3 tumour mass introduced 3D bone stroma model at day 8. The AM-3 tumour mass was introduced at day 6 of 3D bone stroma model. Volcano plot shows under-expressed, unchanged and over-expressed genes. The table represents > 3.5-fold under-expressed gene. Horizontal line p-value threshold (0.05). One-Way ANOVA, Dunnet’s Post Hoc; p-values 0.05 = *, 0.005 = **, 0.0005 = *** and 0.00005 = ****.

In order to understand the interaction between ameloblastoma and its surrounding bone stroma, we utilised the compartmentalised tumouroid model. Here ameloblastoma tumour masses were introduced on different days to active bone forming stromal compartments. We established that bone formation occurs day 9 in 3D, tumour masses were cast on top of the 3D bone stroma before (day 6 of culture) and after bone nodules formation (day 9 of culture). Introduction of an ameloblastoma tumour mass at day 6 completely inhibited bone nodule formation and resulted in limited mineral deposition by osteoblasts compared to 3D bone control cultures (Fig. 5a). This finding was also verified by assessing *ALPL* expression. For each set-ups, the pH was measured continuously and all measurements were pH > 7.1, deemed critical for bone formation^57^. To prevent nutrient depletion, media with BMA was doubled for all cultures. Acellular tumour masses were also introduced in control cultures to confirm that the inhibition of bone formation by osteoblasts was not induced by the tumour mass introduction method. Introduction of control acellular (empty) masses did not cause any decrease in *ALPL* levels. Introduction of AM-1 and AM-3 down-regulated *ALPL* fourfold by day 8 (p < 0.0001). Control osteosarcoma MG-63 tumour masses were introduced to the 3D bone stroma and *ALPL* levels were not downregulated as much as AM-1 tumour mass introduced and AM-3 tumour mass introduced 3D bone stroma models (p = 0.004 and p = 0.005 respectively). *TNFSF11* expression by the osteoblasts in the 3D bone stroma model was upregulated by 6.3-fold by the introduction of AM-1 tumour mass compared to the control (3D bone stroma model) (p = 0.05) (Fig. 5a).

Both AM-1 and AM-3 tumour masses introduced on day 9 did not reduce the sizes of existing bone nodules, however significantly limited further bone formation in terms of number and size of nodules (p < 0.0001) compared to the control 3D bone stroma, evident after day 11. At day 21, the engineered 3D bone stroma model had 15.0 $\pm$ 1.5 bone nodules, where AM-1 tumour mass introduced cultures had 4.0 $\pm$ 0.6 and the AM-3 tumour mass introduced had 2.3 $\pm$ 0.9 bone nodules (p < 0.0001) (Fig. 5b). The bone nodule sizes were 3 times and 5 times (respectively) smaller compared to control 3D bone stroma (p < 0.0001) (Fig. 5b).

### AM-3 cells inhibit osteoblast differentiation

The RT^2^ Profiler PCR Array (Qiagen) was used to screen 84 osteogenesis genes in osteoblasts from AM tumour mass introduced 3D bone stroma where AM tumour masses introduced (AM tumour mass + 3D bone) in comparison to ones in control 3D bone stroma (Fig. 5c).

Introduction of an AM-3 tumour mass downregulated 30 genes and upregulated 1 gene in the osteoblasts. *ALPL* and bone development *COL2A1*^58^, were under-expressed by 5.5-fold (p = 0.003) and 4.2-fold (p = 0.01) respectively. A differentiation factor *GDF10* was under-expressed by 17.8-fold (p = 0.0007), which indicated strong inhibition of osteoblast differentiation by AM-3 cells. AM-3 cells caused a significant reduction in osteoblast differentiation by downregulating differentiation markers including *RUNX2* (p = 0.01), *CD36* (p = 0.0007) and *FGFR2* (p = 0.03). *TGF* $\beta$ *3* (p = 0.02) was under-expressed by 4.03, which was associated with decreased ECM development and mineralisation^59^.

Similar to AM-3 cells, AM-1 cells also did not impact most osteogenesis genes but mostly downregulated ECM and bone development genes such as *ALPL* and *COL2A1,* as well as differentiation genes such as *GDF10*. However, no significant difference was found between the control group and AM-1 tumour mass + 3D bone group (Supplementary Fig. 5).

## Discussion

This work provided three main novel findings. We successfully developed and cultured ameloblastoma cell lines within a biomimetic 3D tumouroid model, which accurately mimicked native subtype cell morphology. Then we established an active bone-forming stromal model in 3D, which formed extensive biomimetic bone nodules. By using the compartmentalised tumouroid model, we introduced the ameloblastoma tumour mass to the stroma and measured the direct effect on the bone forming capabilities of osteoblasts and studied the interaction between ameloblastoma and its native bone stroma. We showed direct inhibition of bone nodule formation by osteoblasts when ameloblastoma is present.

Our aim was to engineer a biomimetic tumour microenvironment by culturing an ameloblastoma tumouroids within dense, collagen I based extracellular matrix, representative of normal tumour tissue^27^. We tested whether existing ameloblastoma cell lines from the two most common histopathological types (AM-1 and AM-3) would represent the morphology and histopathological phenotype of subtypes in patients’ tumour. We were able to compare the growth and invasion pattern differences between the plexiform and follicular cell lines. AM-1 cells and MG-63 cells invaded far greater distances with a cell sheet morphology, in direct comparison to AM-3 cells, which invaded lesser distance and exclusively as invasive spheroid bodies. The spheroid bodies were also associated with lower metabolic activity, which might be due to cell apoptosis in the core of spheroids^60^ Additionally, we have previously shown that highly invasive colorectal cancer cell lines invaded as cell sheets, compared to less invasive cell lines, which retained spheroid bodies within tumouroids^48^. These invasion patterns matched the histopathological properties of each type. The follicular ameloblastoma type forms small islands, that can be cystic^50^, and we observed similar morphology in AM-3 tumouroid models. The plexiform ameloblastoma type branches to form anastomosing cords^50^ in double layer cells, which we observed in AM-1 tumouroid models. We observed biomimetic histopathological phenotype of the ameloblastoma tumouroids, representative of patient samples of both subtypes. Currently, histopathological subtypes are known to have no effect on prognosis and plexiform and follicular types can be found together^61^. However, it might be important to investigate the link between subtypes and aggressiveness of the disease.

We showed that there was higher and earlier production of bone resorption proteins in 3D ameloblastoma tumouroids compared to 2D culture. AM-3 cells presented higher expression of MMP-2, MMP-9, RANK and RANKL compared to AM-1 cells. This observation may be explained by the distinctive spheroid bodies formed in the AM-3 subtype, generating an autocrine effect, which ultimately enhanced protein expression.

We bio-engineered an active bone-forming stroma with live osteoblasts, to study the interaction between ameloblastoma and native bone stroma. In vitro* bone nodule formation* by rat calvarial cells and human progenitor cells is well-established in 2D and here we used similar osteodifferentiation methodology to form bone nodules in 3D^38,62^. Our novel active bone forming model depended on high collagen density and stiffness, which is a biomimetic for tissues in vivo^63^. Although extensively used, soft collagen hydrogen matrices have a high water content^64^ and they failed to mimic the native stiffness and density of collagen found within the bones. Resident osteoblasts potentially clustered quicker in the stiff matrix than soft matrix, due to increased cellular and matrix density. We characterised this 3D bone stroma extensively, and did not limit the study to mineralisation assessments via ALP assay or alizarin red staining^34^. The alizarin red stain or von Kossa stain indicated mineralisation (dystrophic calcification)^65^. We used TEM to show mineral deposition within the dense collagen matrix. Raman spectroscopy verified the presence of mineral and matrix components^66^ From the Raman analysis, the mineral to matrix ratio was similar but higher than tissue engineered bone as reported by *Gentleman *et al*.,*^42^. This finding is likely to be due to the methodologies used for background subtraction, where we subtracted the collagen background necessary for 3D culture. There may also be difference in structure of bone formed in 3D compared to 2D. Nano-CT analysis enabled tracking of rapid bone formation and detail around the bone structure. We confirmed the expression of early and late gene markers for bone formation. The extensive characterisation methods indicated mineralised ECM as well as normal bone structure. We utilised the 3D bone stroma model for ameloblastoma research but hope this model will be used a range of bone-associated diseases.

Due to the lack of an appropriate 3D model to date that would mimic the bone tumour microenvironment, the studies were limited to the action of AM to mineralisation rather than bone formation. For example, the mouse pre-osteoblastic cell line KUSA/1 had lower ALP activity when cultured in AM-1 conditioned media^67^. In this study, we introduced ameloblastoma tumour masses to 3D bone stroma and reported the direct inhibition of bone nodule formation. The model allowed for a temporal picture to be formed along the bone formation timeline. Furthermore, we tested the impact on the bone stroma of introduction of acellular collagen mass to test the impact on diffusion of nutrients and pH within the compartmentalised model. Ameloblastoma cells blocked bone nodule formation completely if they were added to the culture before day 9. After day 9, ameloblastoma cells did not stop the growth of nodules in the process of developing but limited their size and number. The gene work confirmed that ameloblastoma has specific osteogenesis targets mainly focused on stopping osteoblasts from differentiating, which is a critical step in bone formation.

Ameloblastoma cells caused osteoblasts to increase their *TNFSF11* (RANKL) expression. This finding deepens our understanding of the mechanism by which ameloblastoma cells indirectly activate osteoclasts through osteoblasts, ultimately leading to bone resorption. Both cell lines expressed membrane-bound RANK, which could be one of the factors for the upregulation of *TNFSF11* in osteoblasts. We report the expression of membrane-bound RANKL, however further investigation must see whether ameloblastoma cells can also release RANKL^30^, which would mean a direct activation of osteoclasts is also at play.

These findings will help in the development of patient-specific humanised models of ameloblastoma to test potential therapeutics with the aim of personalised healthcare. Future work should include the introduction of osteoclasts to this model to understand whether ameloblastoma-induced activation of osteoclasts can resorb bone.

## Supplementary Information

Supplementary Video 1.

Supplementary Information 1.
