# Supplementary material for: Bioengineering the ameloblastoma tumour to study its effect on bone nodule formation
Source: Sci Rep. 2021 Dec 16;11:24088. doi: 10.1038/s41598-021-03484-5 (PMC8677805; doi:10.1038/s41598-021-03484-5)
Supplement: Supplementary file 2 — Supplementary Information 1. [file 41598_2021_3484_MOESM2_ESM.docx]

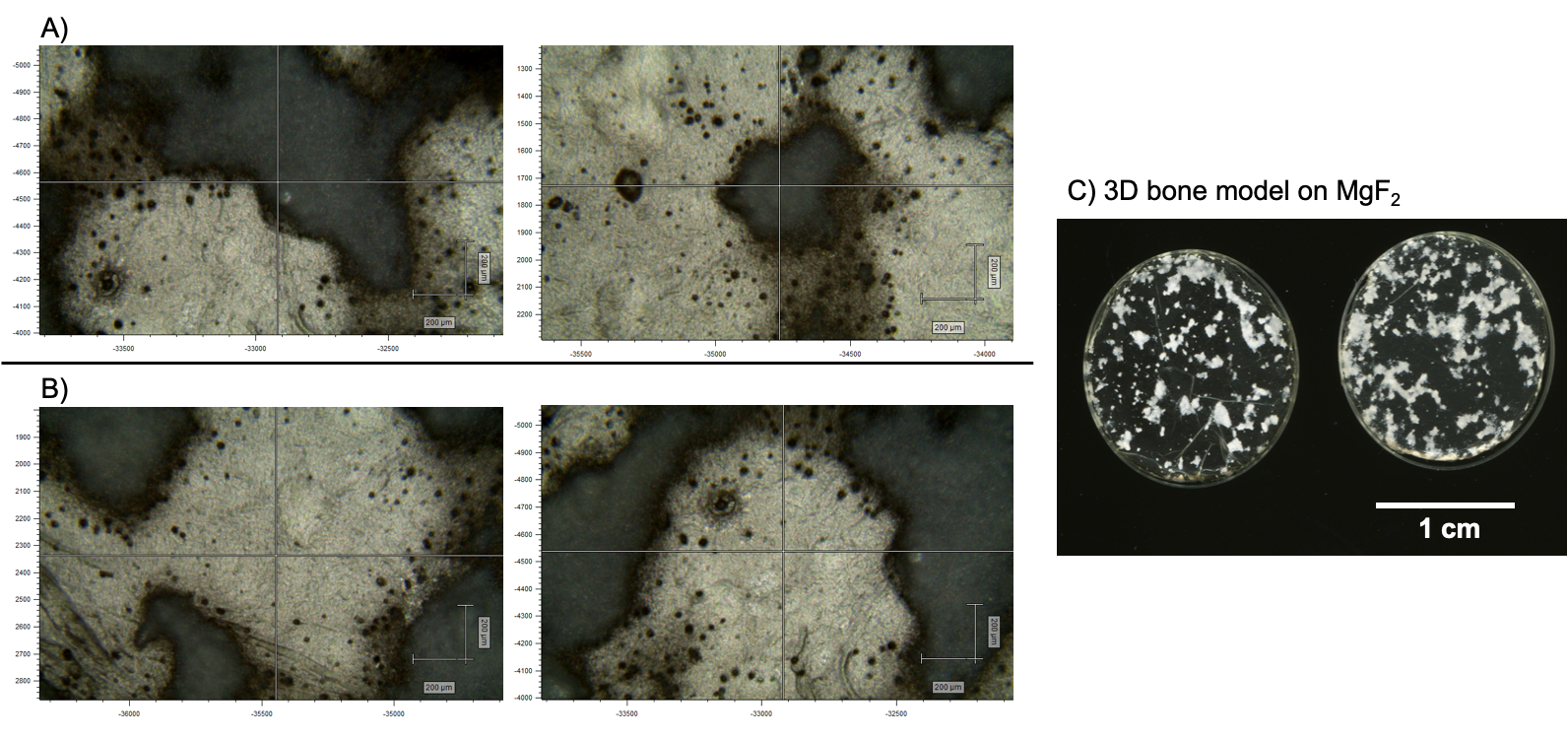


***Supplementary Figure 1: Raman spectra measurements of different focal points for the compositional analysis of the bone nodules in 3D bone stroma model at day 21.*** *A) Focused on top of the bone nodules. B) Focused on the background collagen (20x). C) 3D bone model samples air-dried on MgF_2_ discs.*

| *Gene*  *Primer Pair* | Product Size (bp) | Number of Standards | Efficiency |
| --- | --- | --- | --- |
| ***Rat Primers*** | | | |
| *GAPDH (Glyceraldehyde 3-phosphate dehydrogenase)*  *F’ TTACCAGGGCTGCCTTCTCTTG*  *R’ CACCATCTTCCAGGAGCGAGAT* | 191 | 4 | 97.20% |
| *ALP1 (Alkaline Phosphatase, biomineralisation associated) (AKP2)*  *F' GAGATGGTATGGGCGTCTCCAC*  *R' GCGTTGGTGTTGTACGTCTTGG* | 139 | 3 | 101.70% |
| *E11 (Podoplanin (Pdpn))*  *F' GAGATAACGCAGGCGGTGAAAC*  *R' GATCCCTCCAATGAAGCCAATG* | 116 | 5 | 95.00% |
| *Tumour necrosis factor (TNF superfamily, member 11)*  *F'CTGTACGCCAACATTTGCTTC*  *R'CCCTGACCAGTTCTTAGTGCTC* | 153 | 3 | 96.30% |
| ***Human Primers*** | | | |
| *HPRT1 (Hypoxanthine-guanine phosphoribosyltransferase)*  *F' CCTGGCGTCGTGATTAGTGATG*  *R' TGAGCACACAGAGGGCTACAATG* | 190 | 4 | 90.20% |
| *MMP-2 (Matrix Matalloproteinase-2)*  *F' TCCAAGTCTGGAGCGATGTGAC*  *R' TGAGCCAGGAGTCCGTCCTTAC* | 136 | 3 | 101.40% |
| *TNFRSF11A (TNF Receptor Superfamily Member 11a)*  *F' TGGGACGGTGCTGTAACAAATG*  *R' GGGCCTTGCCTGTATCACAAAC* | 165 | 4 | 95.50% |

***Supplementary Table 1: Rat and human primer pairs and the efficiency test results with the number of test of standards and percentage of efficiency.***

| **Primer3Plus** | **mfold** |
| --- | --- |
| Product Size: Min-100 bp, Opt-120 bp, Max-200 bp  Primer Size: Min-20 bp, Opt-22bp, Max-25-bp  Primer Temperature: (For Ta to be 60^o^C) Min-58^o^C, Opt-60^o^C, Max-65^o^C  Max Self Complementary: 5.00  Max 3’Self Complementarity: 3.00  Max Poly-X: 3  GC Clamp: 1 | Temperature: 60^o^C  Ionic Conditions of SYRB in mM: 50 Na^+^, 3 MG2^+^ |

***Supplementary Table 2: Primer Conditions.***

| *Acvr1* | Activin A receptor, type I |
| --- | --- |
| *Ahsg* | Alpha-2-HS-glycoprotein |
| *Alpl* | Alkaline phosphatase, liver, bone, kidney |
| *Anxa 5* | Annexin A5 |
| *Bglap* | Bone gamma-carboxyglutamate (gla) protein |
| *Bgn* | Biglycan |
| *Bmp1* | Bone morphogenic protein 1 |
| *Bmp2* | Bone morphogenic protein 2 |
| *Bmp3* | Bone morphogenic protein 3 |
| *Bmp 4* | Bone morphogenic protein 4 |
| *Bmp 5* | Bone morphogenic protein 5 |
| *Bmp 6* | Bone morphogenic protein 6 |
| *Bmp 7* | Bone morphogenic protein 7 |
| *Bmpr1a* | Bone morphogenic protein receptor, type IA |
| *Bmpr1b* | Bone morphogenic protein receptor, type IB |
| *Bmpr2* | Bone morphogenic protein receptor, type II (serine/threonine kinase) |
| *Cd36* | CD36 molecule (thrombospondin receptor) |
| *Cdh11* | Cadherin 11 |
| *Chdr* | Chordin |
| *Col10a1* | Collagen, type X, alpha 1 |
| *Col14a1* | Collagen, type XIV, alpha 1 |
| *Col1a1* | Collagen, type I, alpha 1 |
| *Col1a2* | Collagen, type I, alpha 2 |
| *Col2a1* | Collagen, type II, alpha 1 |
| *Col3a1* | Collagen, type III, alpha 1 |
| *Col4a1* | Collagen, type IV, alpha 1 |
| *Col5a1* | Collagen, type V, alpha 1 |
| *Col6a1* | Collagen, type VI, alpha 1 |
| *Comp* | Cartilage oligomeric matrix protein |
| *Csf1* | Colony stimulating factor 1 (macrophage) |
| *Csf2* | Colony stimulating factor 2 (granulocyte macrophage) |
| *Csf3* | Colony stimulating factor 3 (granulocyte) |
| *Crsk* | Cathepsin K |
| *Dlx5* | Distal-less homeobox 5 |
| *Egf* | Epidermal growth factor |
| *Fgf1* | Fibroblast growth factor 1 |
| *Fgf2* | Fibroblast growth factor 2 |
| *Fgfr1* | Fibroblast growth factor receptor 1 |
| *Fgfr2* | Fibroblast growth factor receptor 2 |
| *Flt1* | Fms-related tyrosine kinase 1 |
| *Fn1* | Fibronectin 1 |
| *Gdf10* | Growth differentiation factor 10 |
| *Gli1* | GLI family zinc finger |
| *Icam1* | Intercellular adhesion molecule 1 |
| *Igf1* | Insulin-like growth factor 1 |
| *Igf1r* | Insulin-like growth factor 1 receptor |
| *Ihh* | Indian hedgehog |
| *Itga2* | Integrin, alpha 2 |
| *Itga3* | Integrin, alpha 3 |
| *Itgam* | Integrin, alpha M |
| *Itgav* | Integrin. Alpha V |
| *Itgb1* | Integrin, beta 1 |
| *Mmp10* | Matrix metallopeptidase 10 |
| *Mmp2* | Matrix metallopeptidase 2 |
| *Mmp8* | Matrix metallopeptidase 8 |
| *Mmp9* | Matrix metallopeptidase 9 |
| *Nfkb1* | Nuclear factor of kappa light polypeptide gene enhancer in B-cells |
| *Nog* | Noggin |
| *Pdgfa* | Platelet-derived growth factor alpha polypeptide |
| *Phex* | Phosphate regulating endopeptidase homolog, X-linked |
| *Runx2* | Runt-related transcription factor 2 |
| *Serpinh1* | Serine (or cysteine) peptidase inhibitor, clade H, member 1 |
| *Smad1* | SMAD family member 1 |
| *Smad2* | SMAD family member 2 |
| *Smad3* | SMAD family member 3 |
| *Smad4* | SMAD family member 4 |
| *Smad5* | SMAD family member 5 |
| *Sost* | Sclerostin |
| *Sox9* | SRY-box containing gene 9 |
| *Sp7* | Sp7 transcription factor |
| *Spp1* | Secreted phosphoprotein 1 |
| *Tgfb1* | Transforming growth factor, beta 1 |
| *Tgfb2* | Transforming growth factor, beta 2 |
| *Tgfb3* | Transforming growth factor, beta 3 |
| *Tgfbr1* | Transforming growth factor, beta receptor 1 |
| *Tgfbr2* | Transforming growth factor, beta receptor 2 |
| *Tgfbr3* | Transforming growth factor, beta receptor 3 |
| *Tnf* | Tumour necrosis factor (TNF superfamily, member 2) |
| *Tnfsf11* | Tumour necrosis factor (TNF superfamily, member 11) |
| *Twist1* | Twist homolog 1 (Drosophila) |
| *Vcam1* | Vascular cell adhesion molecule 1 |
| *Vdr* | Vitamin D (1,25-dihydroxyvitamin D3) receptor |
| *Vegfa* | Vascular endothelial growth factor A |
| *Vegfb* | Vascular endothelial growth factor B |
| *Actb* | Actin, beta |
| *B2m* | Beta-2 microglobulin |
| *Hprt1* | Hypoxanthine phosphoribosyl transferase 1 |
| *Ldha* | Lactate dehydrogenase A |
| *Rplp1* | Ribosomal protein, large, P1 |
| *Rat Genomic DNA Contamination* |  |
| *Reverse Transcription Control* |  |
| *Reverse Transcription Control* |  |
| *Reverse Transcription Control* |  |
| *Positive PCR Control* |  |
| *Positive PCR Control* |  |
| *Positive PCR Control* |  |

**Supplementary Table 3:** Gene list of RT2 Profiler PCR Array (96-well format) Rat Osteogenesis Cat. No. 330231 PARN-026ZA (Qiagen).

*
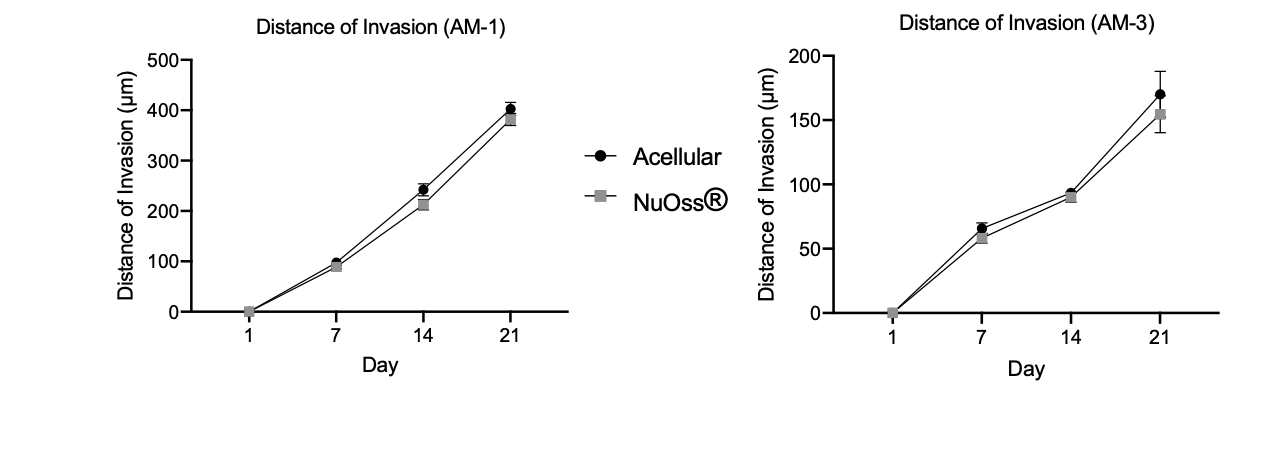
*

***Supplementary Figure 2: Distance of Invasion (of AM-1 and AM-3 tumouroids with acellular and NuOss stroma.***


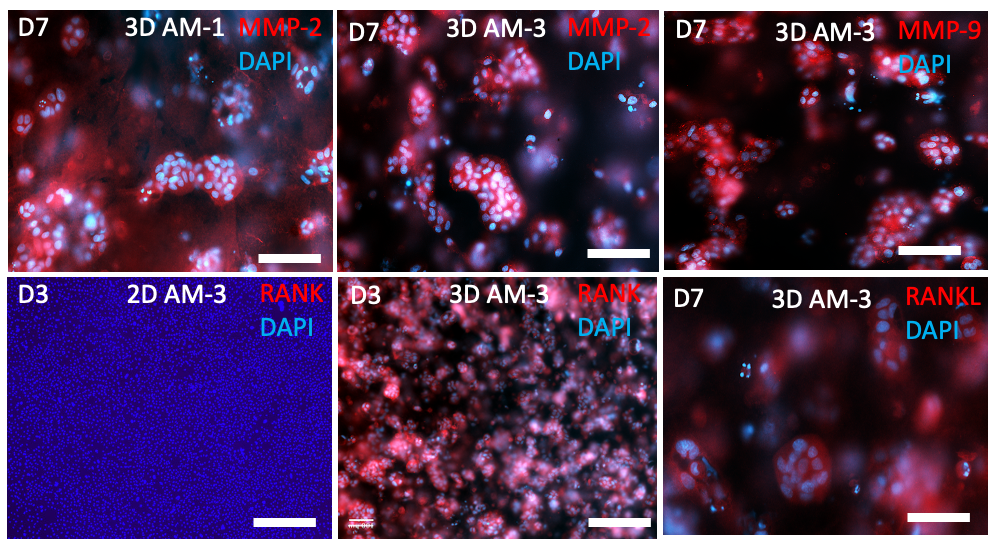


***Supplementary Figure 3: Detection of membrane-bound bone resorption markers; MMP-2, MMP-9, RANK, and RANKL in AM-1 and AM-3 cells in 2D and 3D tumouroids at day 3 (D3) and day 7 (D7).***

***
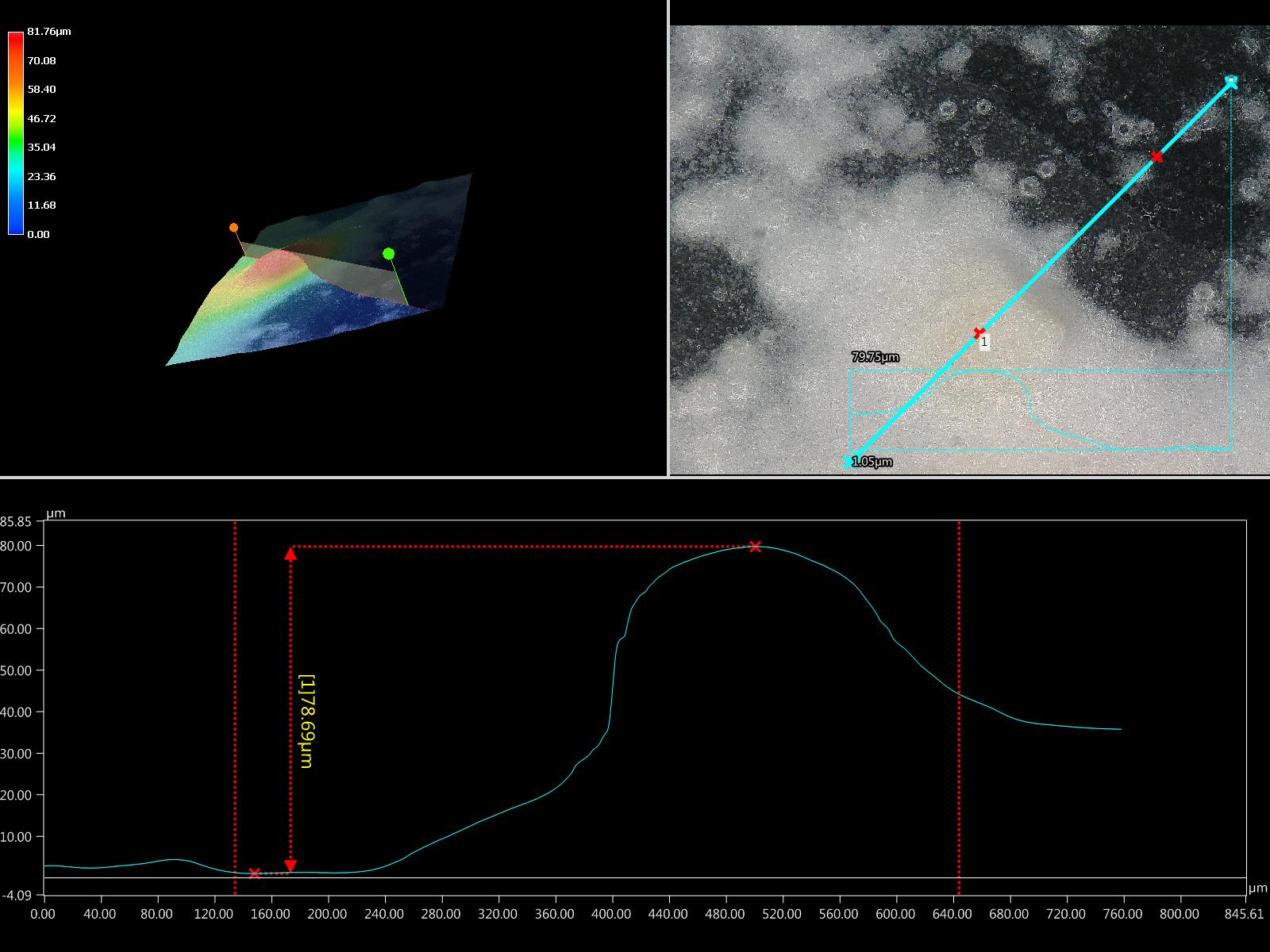
***

***Supplementary Figure 4: Height measurement of day 21 bone nodules from top of the nodule relative to collagen layer. Keyence VHX-7000 Digital Microscope (Keyence, Osaka, Japan).***


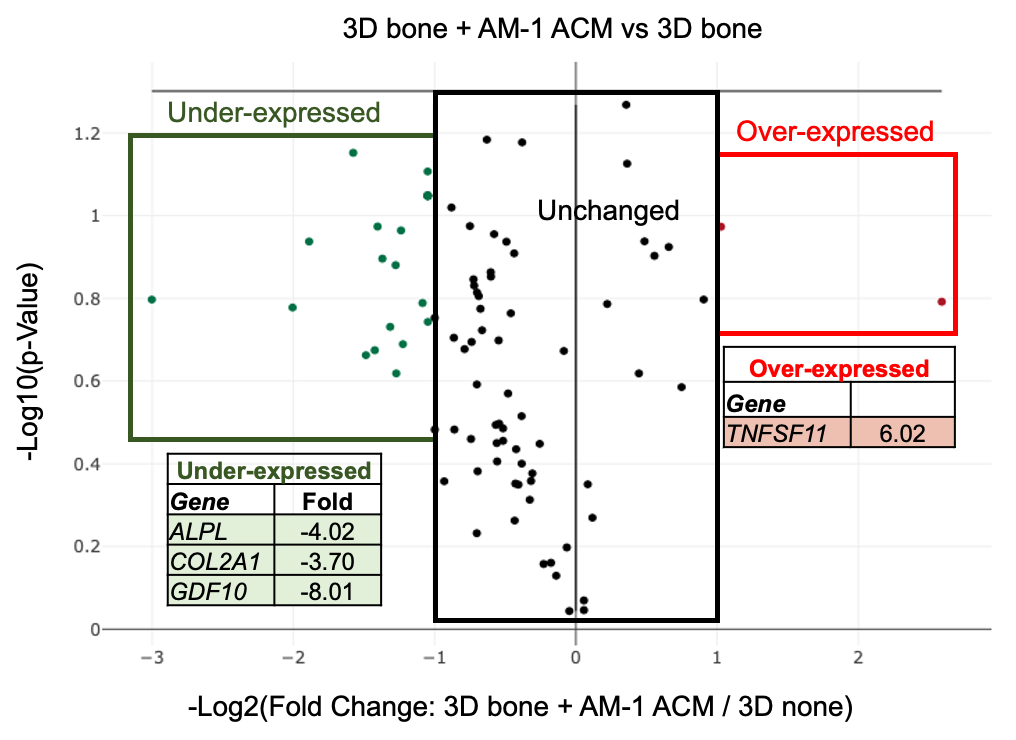


***Supplementary Figure 5: RT2 Profiler PCR Array was conducted to screen osteogenesis gene of osteoblasts in the 3D bone stroma model and in AM-1 ACM introduced 3D bone stroma model at day 8.*** *The AM-1 ACM was introduced at day 6 of 3D bone stroma model. Volcano plot shows under-expressed, unchanged and over-expressed genes. The table represents >3.5-fold under-expressed gene. Horizontal line p-value threshold (0.05). One-Way ANOVA, Dunnet’s Post Hoc; p-values 0.05 = *, 0.005 = **, 0.0005 = *** and 0.00005 = ****.*
